# Onset of Work Restriction in Employed Adults with Lower Limb Joint Pain: Individual Factors and Area-Level Socioeconomic Conditions

**DOI:** 10.1007/s10926-013-9443-z

**Published:** 2013-05-08

**Authors:** Ross Wilkie, Milisa Blagojevic-Bucknall, Kelvin P. Jordan, Glenn Pransky

**Affiliations:** 1 Arthritis Research UK Primary Care Centre, Primary Care Sciences, Keele University, Keele, Staffordshire ST5 5BG UK; 2 Center for Disability Research, Liberty Mutual Research Institute for Safety, 71 Frankland Rd., Hopkinton, MA 01748 USA

**Keywords:** Osteoarthritis, Joint pain, Work disability, Psychosocial deprivation, Socioeconomic factors, Multilevel analyses

## Abstract

*Purpose* To examine individual and area-level socioeconomic factors that predict the onset of work restriction in employed persons with lower limb joint pain. *Methods* Population-based prospective cohort study. Adults were aged 50–59, reported hip, knee, foot pain or a combination and maintained employment through 3 year follow-up (n = 716). Work restriction was measured as inability to participate in work *as desired*. Multi-level logistic regression was used to assess the associations of work restriction onset with baseline factors: health (severity of knee pain/functional limitation, comorbidity, anxiety, depression, cognitive impairment, abnormal weight), demographic socio-economic, environment and area-level employment deprivation. *Results* 108 (15.1 %) reported the onset of work restriction over 3 years. Severe lower limb joint pain and functional limitation, number of affected body sites and area employment deprivation were independently associated with onset. Significant interactions indicated a greater effect of area employment deprivation on older and more depressed workers. *Conclusions* Results suggest that effectively preventing work disability in those with OA will require both condition-specific interventions to decrease pain and maintain function, and providing alternative employment opportunities for those with progressive functional limitations. Results in older workers are particularly concerning, as retirement ages are expected to increase in the general population.

## Introduction

Osteoarthritis is the most common joint condition in adults and globally is the fastest increasing major heath condition [1]. It is recognised as one of the leading and rapidly growing causes of disability [2]. Work restriction is one form of disability and will become more important as adults work to older ages prior to state pension awards, and have greater financial needs resulting from inadequate retirement resources [3]. Its most disabling manifestation (lower limb joint pain) is strongly associated with ageing [4] and with the commonest forms of disability [5–9]. The rise in state pension age raises the likelihood that the prevalence of adults in employment with lower limb joint pain will increase. However the extent to which this will result in greater work restriction among older adults is unclear.

The impact of regional and local social conditions, including neighbourhood deprivation, is increasingly recognised as a determinant of health and its consequences [10–14]. Type of work and educational attainment have been linked to work restriction in adults with osteoarthritis [15–19], but the impact of living conditions and area socioeconomic deprivation is unknown. Older adults with joint pain may be restricted in work because of the characteristics of the area they live in, such as low levels of employment opportunities or poor access to healthcare.

We have previously reported on the frequency and individual risk factors for the onset of any form of participation restriction in all older people in the general population [20, 21]. This paper explores both individual and area-level contextual socioeconomic factors that predict the onset of work restriction in those with lower limb osteoarthritis who remain in work. We focused on persons with lower extremity osteoarthritis symptoms, as it was presumed that these conditions would have an impact on work participation across a wide range of jobs, interfering with travel to and from work, and within the workplace. In contrast, different upper extremity conditions could have a highly variable effect—for example, shoulder disorders might have a significant effect in manual labourers, but not impact clerical workers; hand osteoarthritis could have an opposite impact on work ability.

## Methods

### Study Design and Participants

The study used data from the North Staffordshire Osteoarthritis project; a population-based prospective cohort study [22]. All individuals aged 50 years and over registered with eight general practices were mailed a baseline questionnaire, in 2002, that collected data on health, individual socio-demographic factors and pain, and follow-up questionnaires 3 years later. Reminders were sent to non-responders 2 and 4 weeks after the initial mailing. The North Staffordshire Local Research Ethics Committee approved this study; all participants gave written consent to participate.

For this study we selected a cohort who (1) were aged 50–59 years old at baseline, (2) had hip, knee or foot pain for 1 day or more during the past year at baseline (to indicate lower limb osteoarthritis), (2) indicated that they were in employment at baseline and 3 years and (4) completed the items on work restriction at both time points (n = 716; mean age 54.5 (standard deviation 2.6 years), 54.7 % were female). Compared to subjects who were aged 50–59, had lower limb joint pain, were in employment and free of work restriction at baseline but withdrew, did not respond or had incomplete data at 3 years (n = 651), the participants in this analysis were no more likely to be older (*p* = 0.06), female (*p* = 0.71), have an inadequate income (*p* = 0.51), have better physical (*p* = 0.82) or mental health (0.61) but were more likely to have gone onto further education (*p* = 0.002).

### Data Collection

Work restriction was measured by one item from the Keele Assessment of Participation (KAP) [23]; “During the past 4 weeks, if you work, have you taken part in paid or voluntary work as and when you have wanted?” (all/most/some/a little/none of the time). The reliability and validity of the KAP are adequate for providing estimates of perceived participation restriction in population studies [23]. Three year onset of work restriction was defined as moving from no restriction at baseline (all/most of the time) to work restriction at 3 years (some/a little or none of the time).

The independent variables in the analysis represented lower limb joint pain and functional limitation, comorbidities, age, gender, individual socio-economic, environmental factors and area-level socio-economic factors.

Lower limb pain and functional limitation were measured using the Western Ontario and McMaster Universities Osteoarthrits Index (WOMAC) [24] for those with hip and knee pain and the Foot Disability Index [25] for those with foot pain. The WOMAC offers a five point ordinal scale (none/mild/moderate/severe/extreme) to measure the amount of pain experienced during five tasks and the amount of physical limitation in seventeen tasks. The Foot and Disability Index consists of 19 items designed to measure the effects of foot pain on physical activities. Responses are on a three-point scale (none of the time/on some days/on most or every day). Respondents were categorised as having severe lower limb joint pain and functional limitation if either (1) those with hip or knee pain indicated “severe” or “extreme” pain in any of the five pain items or limitation on the sixteen items for physical functioning or (2) those with foot pain indicated foot pain “on most or every day” on any of the items of the functional limitation or pain intensity constructs (i.e. items 1–11, 14–17).

The co-morbidities included in this analysis were previously found to be associated with participation restriction in at least one aspect [26]. These were: musculoskeletal comorbidity (number of affected body sites), number of self-reported health conditions, anxiety, depression, abnormal weight, and cognitive impairment.

A pain manikin was included to measure musculoskeletal comorbidity. The pain manikin allowed responders who had body pain over the previous 4 weeks to shade their painful body sites (0–44) on a full body manikin (front and back views). The number of shaded body sites was calculated and responses categorised into groups with approximately equal numbers of responders (0, 1–6 affected body sites, 7–44 affected body sites) [20]. Number of health conditions was a simple count of the presence of four self-reported health conditions common in older adults (chest problems, heart problems, diabetes and raised blood pressure). Anxiety and depression during the previous week were measured using the hospital anxiety and depression scale (HADS)—raw scores were calculated and used to categorise individuals as non-cases (0–7) and possible/probable cases (8–21) [27]. Cognitive impairment was measured using the Cognitive and Alertness behaviour subscale of the Functional Limitations Profile—raw scores were categorised to no impairment (score of 0) and cognitive impairment (score > 0) [28]. Body mass index (BMI) was calculated from self-reported height and weight—responders were categorised into standard BMI groups (1) normal weight (BMI 20–24.9 kg m^−2^), (2) underweight (BMI <20 kg m^−2^), (3) overweight (BMI 25–29.9 kg m^−2^) and (4) obesity (BMI ≥30 kg m^−2^) [29].

Individual socio-economic characteristics included were those previously found to be associated with participation restriction in the general population [26]: occupational class (manual/non-manual) [30, 31], educational attainment (those who finished their education on leaving school/those who went onto further education such as college or university) and perceived adequacy of income (adequate/inadequate) [32].

Demographic details collected were age, gender, living arrangement (live alone/live with someone), and social networks (measured with the Berkman–Syme Social Network Index [33]).

Data were collected by single items for three environmental factors relevant to work restriction: one to measure if responders required assistance or aids (i.e. “During the past 4 weeks have you required the assistance of others or aids to move around outside your home?”), one to measure access to transportation (i.e. “Do you have access to a car or public transport when you personally need it?”), and one to measure access to health care “Do you have good access to a GP or chemist?”). These items had a simple yes/no response option.

### Area-Level Socio-Economic Factors

The development of the Index of Multiple Deprivation (IMD) 2004 [34] for England has meant that seven specific socio-economic features of local areas (income, employment, health, education/skills/training, housing, crime, environment) can now be investigated for their effect on an individual’s health. We focused particularly on local area employment deprivation because of its relevance to work. Employment deprivation is conceptualised as involuntary exclusion from the labour market and the more working adults there are in an area that are unemployed, seeking work or on incapacity benefit the greater the employment deprivation, and in a sense this is a proxy for job opportunities and employment in the local area. By focusing on this we could examine if there was a link between good access to job opportunities and the onset of work restriction. The other six domains were included as putative confounders in the multivariate analysis. The index is based geographically at the lower level super output area (SOA) of which there are 32,482 in England with a mean population of 1,500. Subjects are allocated to a SOA based on their postcode. For each domain and for the combined scale, SOAs are ranked from 1 (most deprived) to 32,482 (least deprived). The SOAs from which the subjects in this study were drawn were split into tertiles for each domain of deprivation, the lowest one consisting of most deprived participants and the highest of least deprived participants.

### Statistical Analysis

The frequency of onset of work restriction within each level of area employment deprivation (least, mid, most) was determined. Two level logistic multilevel modelling was then used to examine the associations of individual and area-level variables with onset of work restriction. Prior to the examination of associations, the variance components model (i.e. with no explanatory variables included) was derived, to assess the amount of variation in onset of work restriction that was at the area-level compared to that between individual subjects. The variation at area-level was calculated using the variance partition coefficient defined as ${{\sigma_{u0}^{2} } \mathord{\left/ {\vphantom {{\sigma_{u0}^{2} } {\left( {\sigma_{u0}^{2} \; + \;3.29} \right)}}} \right. \kern-0pt} {\left( {\sigma_{u0}^{2} \; + \;3.29} \right)}}$ where $\sigma_{u0}^{2}$ is the variance of the area-level random effect [35]. The unadjusted associations of individual health, demographic, socio-economic and environmental factors with work restriction onset were then assessed.

The independent effect of each health, demographic, individual socio-economic and environmental factor and area employment deprivation on work restriction onset was then assessed over three stages with reference to the conceptual model of the International Classification of Functioning Disability and Health [36]. In the first stage the “health” model was derived: all health factors were entered simultaneously into the model with age and gender as potential confounders. In the second stage a full multivariate model was derived: all variables significant at 5 % level or with OR >1.3 or <0.77 in the “health” model were included in the model together with the individual socio-economic variables significant in the unadjusted analysis or with OR >1.30 or <0.77 [37]. In the third stage, due to correlation between area-level domains, each of the seven domains was added separately, adjusting for health, demographic and individual socio-economic factors in the multivariate model in stage 2. Associations are summarized by odds ratios with 95 % CIs.

Interaction terms were added to the multivariate model separately. First, we considered the potential for interaction with age to be of prime importance so we added an interaction term between age and (1) severity of joint pain and functional limitations, (2) number of areas affected body sites, (3) depression and then (4) employment deprivation. Second, to examine the role of employment deprivation we added an interaction term between employment deprivation and (1) severity of pain, (2) number of affected body sites and then (3) depression.

Analysis was performed using MLwiN 2.02 [38] via residual iterative generalised least squares with the second order penalised quasi-likelihood approximation.

## Results

Of the 716 included in the analysis, 108 (15.1 %) indicated the onset of work restriction at 3 years. The frequency of onset did not increase with age (*p* = 0.28) or gender (*p* = 0.98). The amount of variation in onset of participation restriction at the area-level accounted for less than 1 % of the total variation.

Severe lower limb joint pain and functional limitation, comorbid pain (7 or more affected body sites), comorbidity, depression, obesity and cognitive impairment were significantly associated with the onset of work restriction at 3 years before adjusting for other factors (Table 1). There were notable associations (ORs >1.30) although not statistically significant between the onset of work restriction and anxiety (OR 1.4; 95 % CI 0.9, 2.1) and overweight (1.2; 0.7, 2.0). In the multivariate analysis, only severe pain and functional limitation, comorbid pain (7 or more affected body sites) and depression retained their significance after adjusting for other health factors and age and gender.

**Table 1:** Associations between the onset of work restriction at 3 years and individual factors: odds ratios with 95 % CIs

	No. of respondents	% restricted	Crude	Health disability model	Multivariate model
			OR (95 % CI)^a^	OR (95 % CI)	OR (95 % CI)
Severity of lower limb joint pain and disability					
Not severe	506	21.1	1	1	1
Severe	210	22.4	2.10 (1.38, 3.20)	1.73 (1.10, 2.71)	1.70 (1.03, 2.83)
Comorbidity					
None	462	12.3	1	1	–
1–4	254	20.1	1.79 (1.18, 2.70)	1.28 (0.81, 2.02)	
Painful areas shaded on manikin^a^					
0	129	7.0	1	1	1
1–6	335	11.9	1.81 (0.85, 3.84)	1.71 (0.79, 3.67)	2.17 (0.92, 5.13)
7–44	238	23.5	4.10 (1.96, 8.60)	3.08 (1.43, 6.64)	3.33 (1.39, 7.94)
Anxiety					
Non-case (0–7)	435	13.6	1	1	–
Possible/probable case (8–21)	273	17.9	1.39 (0.92, 2.11)	0.95 (0.57, 1.56)	
Depression					
Non-case (0–7)	618	13.3	1	1	1
Possible/probable case (8–21)	89	29.2	2.70 (1.62, 4.50)	2.11 (1.13, 3.95)	1.80 (0.88, 3.69)
Body mass index					
Normal (20–24.9 kg m^−2^ **)**	229	12.7	1	1	1
Underweight (<20)	14	14.3	1.15 (0.25, 5.40)	1.87 (0.37, 9.42)	0.89 (0.10, 7.58)
Overweight (25–29.9 kg m^−2^ **)**	307	14.7	1.19 (0.72, 1.96)	1.18 (0.70, 2.01)	1.37 (0.77, 2.44)
Obese (>30 kg m^−2^ **)**	149	20.1	1.74 (1.00, 3.04)	1.36 (0.74, 2.52)	1.34 (0.67, 2.69)
Unknown	17	11.8	0.92 (0.20, 4.23)	0.95 (0.19, 4.67)	0.49 (0.05, 4.44)
Cognitive impairment					
None (0)	452	13.1	1	1	–
Cognitive impairment (0.1–100)	259	18.5	1.52 (1.00, 2.30)	1.09 (0.67, 1.75)	
Age					
50–54	366	13.7	1	1	–
55–59	350	16.6	1.26 (0.83, 1.89)	1.33 (0.86, 2.07)	
Gender					
Male	324	15.1	1	1	–
Female	392	15.1	0.99 (0.66, 1.50)	0.94 (0.60, 1.48)	
Occupational classification					
Non-manual	353	11.6	1	–	1
Manual	351	17.7	1.63 (1.07, 2.50)		1.36 (0.82, 2.26)
Educational attainment					
Further	178	9.0	1	–	1
School only	532	16.9	2.06 (1.18, 3.61)		1.84 (0.92, 3.66)
Adequacy of income					
Adequate	443	13.5	1	–	1
Inadequate	268	17.2	1.32 (0.87, 2.01)		0.99 (0.59, 1.65)
Social networks					
High	217	16.1	1	–	1
Med/high	187	18.2	1.16 (0.69, 1.94)		1.32 (0.73, 2.37)
Med	118	15.3	0.94 (0.50, 1.74)		1.06 (0.54, 2.09)
Low	82	8.5	0.49 (0.21, 1.14)		0.62 (0.25, 1.51)
Living arrangement					
Not alone	620	15.0	1	–	–
Alone	84	16.7	1.13 (0.61, 2.10)		
Access to transport					
Yes	613	14.5	1	–	1
No	98	19.4	1.42 (0.82, 2.45)		0.85 (0.39, 1.84)
Access to health care					
Yes	643	15.2	1	–	–
No	67	14.9	0.98 (0.48, 1.98)		
Requirement for aids/assistance to mobilise					
No	682	14.1	1	–	1
Yes	31	32.3	2.91 (1.33, 6.36)		1.92 (0.68, 5.44)

^a^Odds ratio (95 % CI)

The frequency of onset of work restriction increased with increasing age although this was not significant (13.7 % for those aged 50–54 vs. 16.6 % for those aged 55–59; *p* = 0.28). The strongest unadjusted association occurred between onset and baseline requirement for aids/assistance to mobilize (2.9; 1.3, 6.4). Educational attainment (2.0; 1.2, 3.6) and manual occupation (1.6; 1.1, 2.5) were also significantly associated with onset and there were notable associations between onset and baseline measures of inadequate income (1.3; 0.9, 2.0) and poor access to transport (1.4; 0.8, 2.5). However, none of the individual socio-economic or environmental factors were independently associated with onset.

Onset of work restriction increased with increasing area-level employment deprivation and was independently associated after adjusting for the multivariate model (Table 2) (i.e. mid and most deprived for employment (2.5; 1.3, 4.7 and 2.1; 1.1, 4.1).

**Table 2:** Associations between work restriction onset and area-level employment deprivation status in adults aged 50–59, with lower limb joint pain and who are in employment (n = 716): prevalence and odds ratios with 95 % CIs

Deprivation status	Work restriction		Adjusted OR^a^ (95 % CI)
	No. of respondents	Restricted No. (%)	
Least	233	24 (10.3)	1
Mid	243	42 (17.3)	2.45 (1.27, 4.70)
Most	240	42 (17.5)	2.09 (1.06, 4.13)

^a^Adjusted for all individual level factors in final model (health/disability and socio/economic model); area-level factors entered separately

Focusing on age, the interactions between age and severity of lower limb pain severity, number of affected body sites, depression and employment deprivation were statistically associated with onset of work restriction when adjusted for health risk factors, occupation, education, income and gender (Table 3), indicating a greater effect in older persons. There were notably strong associations between onset and the interaction between age and employment deprivation—those aged 55–59 and living in the mid deprived areas for employment deprivation had four times the odds (3.7; 1.5, 9.3) of becoming restricted in work than those who were aged 50–54 and living in one of the least deprived areas for employment. Interactions between depression and area-level deprivation in employment were also associated with onset of work restriction, with a greater impact of area-level economic deprivation in more depressed persons.

**Table 3:** Interactions significantly associated with onset of work restriction after adjustment for health, demographic, socio-economic, individual and area-level environmental factors in adults aged 50–59, with lower limb joint pain and who are in employment (n = 716)

Interaction variables (n)	Adjusted OR^a^ (95 % CI)
Age * severity of lower limb pain and disability	
50–54 and not severe (270)	1
55–59 and not severe (239)	1.62 (0.86, 3.03)
50–54 and severe (97)	1.84 (0.85, 3.99)
55–59 and severe (114)	2.56 (1.26, 5.21)
Age * number of pain areas	
50–54 and 0 pain areas (61)	0.40 (0.09, 1.87)
55–59 and 0 pain areas (69)	0.82 (0.27, 2.45)
50–54 and 1–6 pain areas (180)	1
55–59 and 1–6 pain areas (156)	1.77 (0.83, 3.79)
50–54 and 7–44 pain areas (120)	1.88 (0.84, 4.19)
55–59 and 7–44 pain areas (120)	2.36 (1.09, 5.08)
Age * depression	
50–54 and not depressed (317)	1
55–59 and not depressed (305)	1.41 (0.82, 2.41)
50–54 and depressed (47)	1.44 (0.52, 4.03)
55–59 and depressed (42)	3.36 (1.27, 8.92)
Age * area-level employment	
50–54 and least deprived (119)	1
50–54 and mid deprived (127)	2.25 (0.85, 5.93)
50–54 and most deprived (121)	2.09 (0.76, 5.73)
55–59 and least deprived (118)	1.45 (0.52, 4.00)
55–59 and mid deprived (116)	3.69 (1.47, 9.27)
55–59 and most deprived (119)	2.92 (1.13, 7.55)
Area-level employment * depression	
Least deprived and not depressed (207)	1
Mid deprived and not depressed (201)	2.23 (1.08, 4.60)
Most deprived and not depressed (214)	2.22 (1.06, 4.62)
Least deprived and depressed (28)	1.60 (0.42, 6.07)
Mid deprived and depressed (36)	6.72 (2.13, 21.20)
Most deprived and depressed (25)	2.61 (0.66, 10.33)

^a^Adjusted for all individual level factors in final model (health/disability and socio/economic model)

## Discussion

This study has examined health and social factors linked to the future onset of work restriction in older adults with lower limb joint pain, with a particular focus on living in an economically-deprived area. Multivariate results indicate that work restriction is driven primarily by the manifestations of pain and related functional limitations. Severity of osteoarthritis has previously been linked with lower productivity [39]. Notably health comorbidity and obesity which are known to add to the impact of osteoarthritis were not linked to work restriction, as these problems may not start to impact on function until older age. Although not statistically significant in the final model, results suggest that depression and educational level (perhaps reflective of occupational factors) may be significant, consistent with findings in other studies.

We have shown a novel finding that living in an area with economic deprivation predicts onset of work restriction in this group of older workers with probable osteoarthritis; the more deprived the area for employment that you live in the more likely you are to develop work restriction, even when you have stable employment. There was also a strong interaction with age, demonstrating that the effect on onset of work restriction of living in an area of employment deprivation increases with increasing age. This result highlights the importance of job opportunities for older adults with lower limb joint pain. Although further research is required to understand the mechanism fully, a lack of job opportunities may prevent individuals from changing job as a method of adapting to job strain caused by joint symptoms, comorbidity or a negative working environment (e.g. low co-worker support) [40]. Or, this might reflect the overall quality of current employment situations, including dimensions such as flexibility at work [41]. This underlines the potential role of non-clinicians and perhaps local and national policy that encourages the provision of employment opportunities in areas where job opportunities are low, as fundamental in preventing the onset of work restriction and ultimately job loss for older adults with lower limb joint pain [42]. The intercorrelation among area socio-economic deprivation factors suggests that one causal pathway could be poor environment leading to poor health, and thus new work restrictions could be a secondary effect [43].

From a clinical perspective, severe lower limb pain and functional limitation was linked with onset of work restriction, and remained so after adjusting for all other health factors, reinforcing the importance of treatment strategies to maximise and preserve functional ability. The interaction analysis highlighted the increasing importance of these factors as workers age. We have previously highlighted the importance of requiring aids for maintaining mobility outside the home and this also appears to play a part in restricting work, or may simply be a marker of persons with the most severe functional impact due to the condition [8].

### Strengths and Limitations

This study focuses on a prevalent problem using a population-based sample, with a unique geographic area-level contextual perspective, and the longitudinal design enables the analysis to focus on the onset of work limitation over time. Restricting analysis to persons continually employed during the observation period implies that answers to the work item relates to the current job, not the ability to find employment in those without work. Thus, it provides an opportunity to examine a potential first step in a gradual progression to health-related job loss in persons with a chronic musculoskeletal condition.

Our sample was derived to allow the analysis to focus on older adults with lower limb joint pain who may be at risk of leaving employment (i.e. they continue in employment but are not working “as and when they want”) before state retirement age. There was an insufficient number (n = 50) of adults who retired early during the three year period to allow a meaningful analysis of the reasons for older adults leaving employment before retirement age. As with any cohort study, non-completion of the work item at both time points and attrition may have led to underestimation of estimates, however based on comparisons between those included in the analysis and those who dropped out, such effects are likely to be small. The area covered by the study is more deprived on health, education, and employment, but with fewer barriers to housing and services, than England as a whole. This may overestimate the strength of associations with area-level employment deprivation but again the effect of this will be small.

Our data collection limits some of our findings. Comorbidity was broadly measured using self-report of a limited number of conditions and may not represent the general disease burden of respondents. The definitions of lower limb joint pain and work restriction were based solely on the previous month at both baseline and 3 years. Therefore, history of work restriction prior to baseline, and exacerbations and recurrences in the 3 years between baseline and follow-up would not be captured. The absence of continual measures of work restrictions limited the ability to identify those who varied considerably over time in having work restrictions; this problem biases our results to the null, and thus the actual effect could be much larger. The question on work restriction is a single item from the KAP and allows individuals to report if they are working as they want. This may not correlate for all individuals with actual restriction but does act as a measure of perceived challenges in work that lead to movement out of employment. Finally, there may be other risk factors at both the individual and area-levels, which may be important but which were not measured in this study. In particular there were no workplace factors included, which might have explained some of the association between work restriction and area-level deprivation; for example, lack of workplace accommodation or co-worker support may be a reason why those with lower extremity pain experience the onset of work restriction.

## Conclusion

Although the majority of older adults continue to work as and when they want despite having lower limb joint pain, a significant group develop work restrictions, in part related to individual and environmental factors. The challenge for an aging society will be to develop strategies to prevent and reduce work restriction that address both types of potential causes, for what will be an increasing number of older adults expected to stay at work.
